# Co-Interactive DNA-Binding between a Novel, Immunophilin-Like Shrimp Protein and VP15 Nucleocapsid Protein of White Spot Syndrome Virus

**DOI:** 10.1371/journal.pone.0025420

**Published:** 2011-09-29

**Authors:** Pakkakul Sangsuriya, Saengchan Senapin, Wei-Pang Huang, Chu-Fang Lo, Timothy W. Flegel

**Affiliations:** 1 Centex Shrimp, Faculty of Science, Mahidol University, Bangkok, Thailand; 2 Department of Biotechnology, Faculty of Science, Mahidol University, Bangkok, Thailand; 3 National Center for Genetic Engineering and Biotechnology (BIOTEC), National Science and Technology Development Agency, Pathum Thani, Thailand; 4 Institute of Zoology, National Taiwan University, Taipei, Taiwan; University of Minnesota, United States of America

## Abstract

White spot syndrome virus (WSSV) is one of the most serious pathogens of penaeid shrimp. Although its genome has been completely characterized, the functions of most of its putative proteins are not yet known. It has been suggested that the major nucleocapsid protein VP15 is involved in packaging of the WSSV genome during virion formation. However, little is known in its relationship with shrimp host cells. Using the yeast two-hybrid approach to screen a shrimp lymphoid organ (LO) cDNA library for proteins that might interact with VP15, a protein named PmFKBP46 was identified. It had high sequence similarity to a 46 kDa-immunophilin called FKBP46 from the lepidopteran *Spodoptera frugiperda* (the fall armyworm). The full length *PmFKBP46* consisted of a 1,257-nucleotide open reading frame with a deduced amino acid sequence of 418 residues containing a putative FKBP-PPIase domain in the C-terminal region. Results from a GST pull-down assay and histological co-localization revealed that VP15 physically interacted with PmFKBP46 and that both proteins shared the same subcellular location in the nucleus. An electrophoretic mobility shift assay indicated that PmFKBP46 possessed DNA-binding activity and functionally co-interacted with VP15 in DNA binding. The overall results suggested that host PmFKBP46 might be involved in genome packaging by viral VP15 during virion assembly.

## Introduction

White spot syndrome virus (WSSV) is the causative agent of white spot disease (WSD) and one of the most serious viral pathogen threats to the shrimp culture industry worldwide. WSD can cause cumulative mortality up to 100% within 3–10 days after infection [1] and has caused dramatic economic losses on farms. WSSV is an ellipsoid to bacilliform, enveloped virus of about 275 nm in length and 120 nm in diameter with a dsDNA genome size of approximately 300 kb (see review by [2], [3]). Three isolates have been completely sequenced, revealing approximately 180–500 predicted open reading frames (ORFs) in the various isolates [4], [5], [6]. Due to its uniqueness among existing virus families, it was re-classified into a new family *Nimaviridae* and genus *Whispovirus* [7]. Genomic and proteomic approaches have been used to gain more insight into the biological and pathological processes of WSSV (see review by [8]). For example, the genomic approach has succeeded in identifying several genes involved in DNA replication, nucleotide metabolism and anti-apoptosis, and the proteomic method has identified more than 40 viral structural proteins (see review by [2], [3]). In addition, studies at the molecular level are being done on host-virus interaction in the hope that a better understanding of the process may lead to new strategies for protection against or treatment for WSD. Recent examples in this area include studies on viral proteins such as VP281 [9], VP28 [10], VP187 [11] and VP53A [12], [13], all of which are among envelope proteins that may be involved in attachment and entry into host cells. Also included are recent studies on WSSV nonstructural proteins that interact with host proteins [14], [15], [16].

Here, we focus on the major nucleocapsid protein VP15, a very basic protein with DNA-binding activity [17] that is thought to be involved in viral genome condensation and packing into the nucleocapsid during viral particle assembly [18], [19], [20]. Using yeast two-hybrid analysis, we identified a novel shrimp protein PmFKBP46 that interacted with VP15. PmFKBP46 showed homology to FK506-binding protein (FKBP) that is found in both prokaryotes and eukaryotes. The interaction between PmFKBP46 and VP15 was subsequently confirmed by both *in vitro* and *in vivo* assays. Computational methods were used to predict the primary and three dimensional (3D) structures of PmFKBP46. Finally, we provide evidence indicating that PmFKBP46 is a DNA-binding protein that may interact with VP15 during the assembly of WSSV viral particles.

## Materials and Methods

### Yeast two-hybrid screening

Yeast two-hybrid library construction and screening were carried out using Matchmaker library construction and screening kits (BD Biosciences). A *P. monodon* lymphoid organ cDNA library fused with the GAL4 activation domain (AD) of pGADT7-rec was obtained from previous work [12]. The 240-bp gene encoding the nucleocapsid protein VP15 (GenBank accession number **AF440570**) was amplified using VP15-F1 and VP15-R1 primers (Table 1) and fused in-frame with the GAL4 DNA binding domain (BD) of pGBKT7. The construct obtained was called BD-VP15, which was then used as bait to screen the library. Before screening, the viral protein was tested for protein expression the in the yeast strain Y187 (*Saccharomyces cerevisiae*) harboring VP15-BD plasmid grown in SD minimal medium lacking tryptophan (SD/-Trp) at 30°C. Crude cell extracts were prepared as described previously [21] and resolved by SDS-PAGE followed by immunoblot assay for detection using anti-c-Myc antibody (BD Biosciences) and goat anti-mouse IgG-HRP conjugate (Sigma). The immunoreactive complex was detected using the chemiluminescence reagent Plus (Perkin Elmer life Sciences). Yeast two-hybrid screening was performed by mating Y187 containing BD-VP15 with AH109 containing the cDNA library according to BD Biosciences' protocol. Positive interactions were indicated by growth on high stringency media lacking adenine, histidine, leucine and tryptophan (SD/-Ade/-His/-Leu/-Trp) and by a blue color-change due to X-α-gal (BD Biosciences) present in the medium. Library plasmids from positive colonies were rescued in *Escherichia coli* DH5α and re-confirmed by yeast cotransformation. In brief, individual, rescued library plasmids and BD-VP15 bait were co-transformed into yeast strain AH109 using the lithium acetate/dimethyl sulfoxide method [22] and growth on SD/-Ade/-His/-Leu/-Trp/X-α-gal was observed. The empty vectors pGADT7 or pGBKT7 were used as negative controls and interaction between murine p53 bait fusion and SV40 prey fusion (BD Biosciences) served as a positive control.

**Table 1:** List of primers used in this study.

Gene	Primer sequence (5′-3′)	RE site	Vector	Remarks
*PmFKBP46*	R1: TATTTTATGGTTTTTAGTTC	NA	NA	5′RACE
	R2: AGATTAGAGTGGAGTTGGGTGGG	NA	NA	5′RACE
	R3: GCTCCTCGCTCACCATACGCCA	NA	NA	5′RACE
	R4: GGGTTTTGTTTTCCACTTTCTTTA	NA	NA	5′RACE
	R5: GTTTGCACTTTATTTCCAGGAGTT	NA	NA	5′RACE
	F: TTCCATATGATGTTTTGGGGTTTGTCAC	*Nde*I	pET-15b	Recombinant protein production
	R: CGCGGATCCTTTTATGGTTTTTAGTTC	*Bam*HI		
*VP15*	F1: GAGGCCAGTGAATTCATGACAAAATACCCCGAG	*Eco*RI	pGBKT7	Yeast two-hybrid assay
	R1: CGATGCCCACCCGGGTGTTAACGCCTTGACTTGCG	*Sma*I		
	F: CGGATCCATGACAAAATACCCCG	*Bam*HI	pGEX-4T-3	Recombinant protein production
	R: AGCGGCCGCAACGCCTTGACTTGCGGGC	*Not*I		

RE site, restriction enzyme site.

NA, not applicable.

### Sequence analysis

Homology searches were performed using BLAST program in the NCBI database (http://www.ncbi.nlm.nih.gov/BLAST/). DNA and protein sequence analyses were carried out using the EXPASY server (http://expasy.org/). Predicted conserved domains and phosphorylation sites were analyzed using ScanProsite [23] and NetPhosK [24], respectively. The 3D-structure of PmFKBP46 was predicted using I-TASSER server [25] and viewed by PyMOL (http://www.pymol.org). The resulting predicted model was assessed by a confidence score (C-score). The structures with C-score values more than 0 were considered reliable results and were used for data interpretation.

### Rapid amplification of 5′ cDNA end (5′RACE) of *PmFKBP46*

Total lymphoid organ RNA was extracted from *P. monodon* using TrizolTM reagent and subjected to two successive 5′RACE reactions using a kit (Roche) to identify the 5′ end of *PmFKBP46* cDNA. Primers used in the RACE assays are listed in Table 1. Gene-specific primers, FKBP-R1, FKBP-R2 and FKBP-R3 were designed based on the partial sequence of *Pm*FKBP46 obtained from the rescued AD library while FKBP-R4 and FKBP-R5 primers were designed based on the sequence results from the first 5′RACE reaction. For the first 5′RACE reaction, shrimp cDNA was synthesized using the FKBP-R1 primer and the resulting product was purified using a PCR purification kit (Qiagen). Then, a poly(A) tail was added to the 3′ end of the cDNA by terminal transferase followed by first-round PCR amplification using FKBP-R2 and oligo(dT)-anchor primers and nested PCR amplification using FKBP-R3 and anchor primers provided with the kit. For the second 5′RACE reaction, the cDNA was synthesized using FKBP-R3 primer followed by poly (A) tail addition as described above. Then the templates obtained were used in the first-round PCR using FKBP-R4 and oligo(dT)-anchor primers and nested PCR using FKBP-R5 and anchor primers. The PCR products were subsequently analyzed by agarose gel electrophoresis and then cloned into the pGEM-T easy vector (Promega) for sequence analysis.

### GST pull-down assay

The coding regions of *PmFKBP46* and *VP15* were amplified with specific primers (Table 1) and cloned into pET-15b (Novagen) and pGEX-4T-3 (GE Healthcare) plasmid, respectively. The empty plasmid pGEX-4T-3 was also used to produce the control GST protein. *E. coli* Rosetta (DE3) harboring pET-15b or pGEX-4T-3 constructs was used to express proteins by induction with 0.5 mM IPTG at 18°C for 16 h. Cells were harvested, resuspended in ice-cold PBS buffer containing 1X protease inhibitor (Roche), and then sonicated on ice. The suspensions were clarified by centrifugation and the supernatant was used for protein purification. Recombinant His-tagged proteins and GST-tagged proteins were purified using Ni-NTA agarose (QIAGEN) and glutathione sepharose 4B (GE Healthcare), respectively according to the suppliers' protocols. The eluates were transferred to a dialysis bag (Spectra/Por 4), dialyzed against PBS and quantified using a Bio-Rad protein assay kit. The purified proteins were analyzed by SDS-PAGE and Coomassie staining. A GST pull-down assay was performed by mixing the purified His-tagged PmFKBP46 protein (PmFKBP46-His) to glutathione sepharose beads coupled with the purified GST-tagged VP15 protein (VP15-GST) or control GST protein. After incubation at 4°C overnight with rotation, beads were washed with PBS buffer and the eluates were resolved by SDS-PAGE followed by immunoblot assays probing with a mouse anti-His antibody (Amersham Biosciences) or an HRP-conjugated anti-GST antibody (Amersham Biosciences) to confirm the presence of the protein complexes. In addition, the pull-down assay was carried out using nuclease-treated purified proteins to confirm direct interaction between PmFKBP46 and VP15 proteins. Each purified protein was mixed with 1 unit of DNase I (Fermentas) and 0.1 µg of RNase A (Fermentas) per 10 µg of proteins and incubated at 37°C for 20 min in order to eliminate any possible contaminating nucleic acids prior to mixing with glutathione sepharose beads as described above.

### PmFKBP46 polyclonal antibody production

The deduced amino acid sequence of PmFKBP46 was used as a template for antigen design and custom peptide synthesis by EZBiolab. The epitope obtained corresponded to PmFKBP46 amino acids 217–231 (KNESQGDKTPKGKAE) and was linked with bovine serum albumin (BSA) (Research organics) using glutaraldehyde. The molar ratio between peptide and BSA was 6∶1. Thus, the linkage reaction contained 0.77 mg of PmFKBP46 peptide, 5 mg of BSA and 0.5% glutaraldehyde in 1 ml of PBS buffer. The reaction was carried out overnight at room temperature with rotation. The contents were subsequently transferred to a dialysis bag (Spectra/Por 4) and dialyzed overnight against distilled water at 4°C, followed by further dialysis overnight against PBS buffer at 4°C. The BSA-linked PmFKBP46 peptide was then sent to Biomedical Technology Research Center, Chiang Mai University for mouse polyclonal antibody production. The antibody obtained was adsorbed with BSA (Research organics) according to general protocols (www.abcam.com/technical) prior to western blot and co-localization assays.

### Specificity test for the custom-made antibody

To test the custom-made antibody against the short peptide sequence of PmFKBP46 (as described above), a western blot was performed using *P. monodon* hemolymph and hemocyte fractions. The hemolymph was withdrawn by mixing with AC-1 anticoagulant solution [26]. Then, hemocytes were collected by centrifugation and homogenized in 50 mM Tris-HCl buffer, pH 8.0 containing 100 mM NaCl and 1 mM phenylmethanesulfonyl fluoride (PMSF). The homogenate was centrifuged at 3,000 x g for 5 min and the supernatant collected for total protein quantification using a Bio-Rad protein assay kit. The total protein from the cell-free hemolymph and hemocyte extracts (approximately 15 µg) were separated by SDS-PAGE and then subjected to immunoblot assay using mouse anti-PmFKBP46 antibody. Positive reactivity was detected using goat anti-mouse IgG-HRP conjugate (Sigma) with TMB substrate solution (Sigma).

### Indirect immunofluorescence assay of PmFKBP46 and VP15 in shrimp hemocytes

The hemolymph from healthy shrimp and WSSV-infected shrimp (72 h post infection) were withdrawn as described previously. Hemocytes were collected by centrifugation and suspended in Leibovitz's L-15 medium (Invitrogen). The hemocyte suspension was then placed on cover glasses in a 4-well plate at approximately 80% confluence and incubated at 27°C for at least 30 min to allow cells to attach. The hemocytes were then washed twice with PBS, fixed with 4% paraformaldehyde in PBS at 4°C for 10 min and permeabilized with acetone at 4°C for 3 min. After blocking with 3% normal goat serum (Jackson ImmunoResearch) overnight at 4°C, the hemocytes were incubated at room temperature for 3 h with rabbit anti-VP15 [27] and with mouse anti-PmFKBP46 antibody. The cells were washed with PBST (0.2% of Tween 20 in PBS) three times (10 min each) and then reacted with FITC-conjugated goat anti-mouse IgG antibody (Sigma) and with Cy3 dye-conjugated goat anti-rabbit IgG antibody (Sigma) at room temperature for 1 h. The nuclei were counterstained with TO-PRO-3 iodide (Molecular Probes) and then extensively washed with PBST. The cover glasses were wet mounted and the slides were viewed using a confocal laser-scanning microscope Model FV1000 (Olympus).

### Electrophoretic mobility shift assay (EMSA)

Electrophoretic mobility shift assays (EMSA) with protein-DNA mixtures were conducted using a protocol modified from [28]. The reactions were performed in 15 µl of binding buffer (20 mM Tris-HCl, pH 8.0 and 200 mM NaCl) containing approximately 300 ng of plasmid DNA (pGEX-5X-1; GE Healthcare) and the appropriate protein to be tested. The purified proteins of PmFKBP46-His and VP15-GST (varying from 0–250 pmol) were individually incubated with the plasmid DNA at 37°C for 1 h before loading onto 0.8% agarose gels. Electrophoresis was performed using TBE buffer followed by ethidium bromide staining and observation by UV transilluminator. Control proteins used in this experiment were the GST protein mentioned above and His-tagged protein purified from plasmid pET-15b containing a partial sequence of shrimp serine proteinase homologue (namely SPH516c) previously reported [29]. To test the regulative effect on DNA-binding activity of both PmFKBP46-His and VP15-GST proteins, the reactions were carried out under 2 different conditions. First, individual proteins or paired proteins were mixed with the plasmid DNA and incubated at 37°C for 1 h. Second, paired proteins were first mixed together at 37°C for 1 h before plasmid DNA was added to the reaction mixture followed by incubation for 1 h. The quantity of protein used in these conditions was adjusted to 1∶5 molar ratio which contained 40 pmol of VP15-GST and 200 pmol of PmFKBP46-His.

## Results

### Identification of VP15-interacting protein by yeast two-hybrid screening

**Figure 1:**
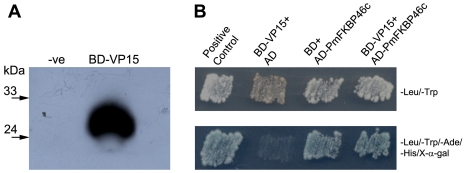
Yeast two-hybrid analysis. (**A**) Photographic film of immunoblot detection of BD-VP15 fusion protein with anti c-Myc antibody, confirming its expression in transformed Y187 compared to un-transformed Y187 (-ve). (**B**) Yeast two-hybrid screen results indicating the presence of BD and AD-plasmids in transformant cells growing on an SD/-Leu/-Trp plate. Putative interaction of BD-VP15 and AD-PmFKBP46c is indicated by growth and blue color on selective medium. The positive control comprised interaction between murine p53 bait fusion and SV40 prey fusion (BD Biosciences). The negative controls comprised yeast cells containing BD-VP15 and empty pGADT7 vector or empty pGBKT7 vector and AD-PmFKBP46c.

For the yeast two-hybrid screen to identify VP15 binding partners from *P. monodon* using the recombinant plasmid BD-VP15 as bait with a *P. monodon* lymphoid organ cDNA library, we first verified that BD-VP15 could be expressed in yeast cells and did not cause self-activation of the reporter genes. The western immunoblot of lysate from BD-VP15-transformed yeast cells yielded an immunoreactive band with the predicted size of approximately 31 kDa using an anti c-Myc antibody, while no detectable band was observed from un-transformed yeast cells (Fig. 1A). In addition, cotransformation with BD-VP15 and pGADT7 did not result in bait self-activation (Fig. 1B, lane BD-VP15+AD). These results indicated that the transformed yeast was suitable for yeast two-hybrid analysis. Subsequent screening results yielded nine independent clones that grew and activated *MEL1* promoter, as indicated by blue colonies on SD/-Ade/-His/-Leu/-Trp/X-α-gal plates (data not shown). Sequence analysis revealed that eight clones had no sequence similarity to any known gene while one clone (namely AD-PmFKBP46c) contained a 623 bp fragment inserted in-frame in pGADT7 that significantly matched sequences in the NCBI database. The deduced amino acid sequence of this clone contained a putative FKBP-PPIase domain (FKBP family of peptidyl-prolyl cis/trans isomerases) and had homology to an immunophilin FKBP46 (46 kDa FK506-binding nuclear protein) of *Spodoptera frugiperda* (GenBank accession number **Q26486**) at 68% identity and 84% similarity. The rescued AD-PmFKBP46c plasmid was cotransformed with BD-VP15 again and interaction indicated by blue colony growth on selective medium was observed as expected (Fig. 1B, lane BD-VP15+AD-PmFKBP46c). However, the rescued AD-PmFKBP46c plasmid seemed to cause self-activation since its combination with empty BD plasmid could also turn blue (Fig. 1B, lane BD+AD-PmFKBP46c). Therefore, the full-length sequence of *PmFKBP46* was required and subsequent confirmation tests using independent assays were investigated (described below).

### Identification and characterization of *PmFKBP46*

**Figure 2:**
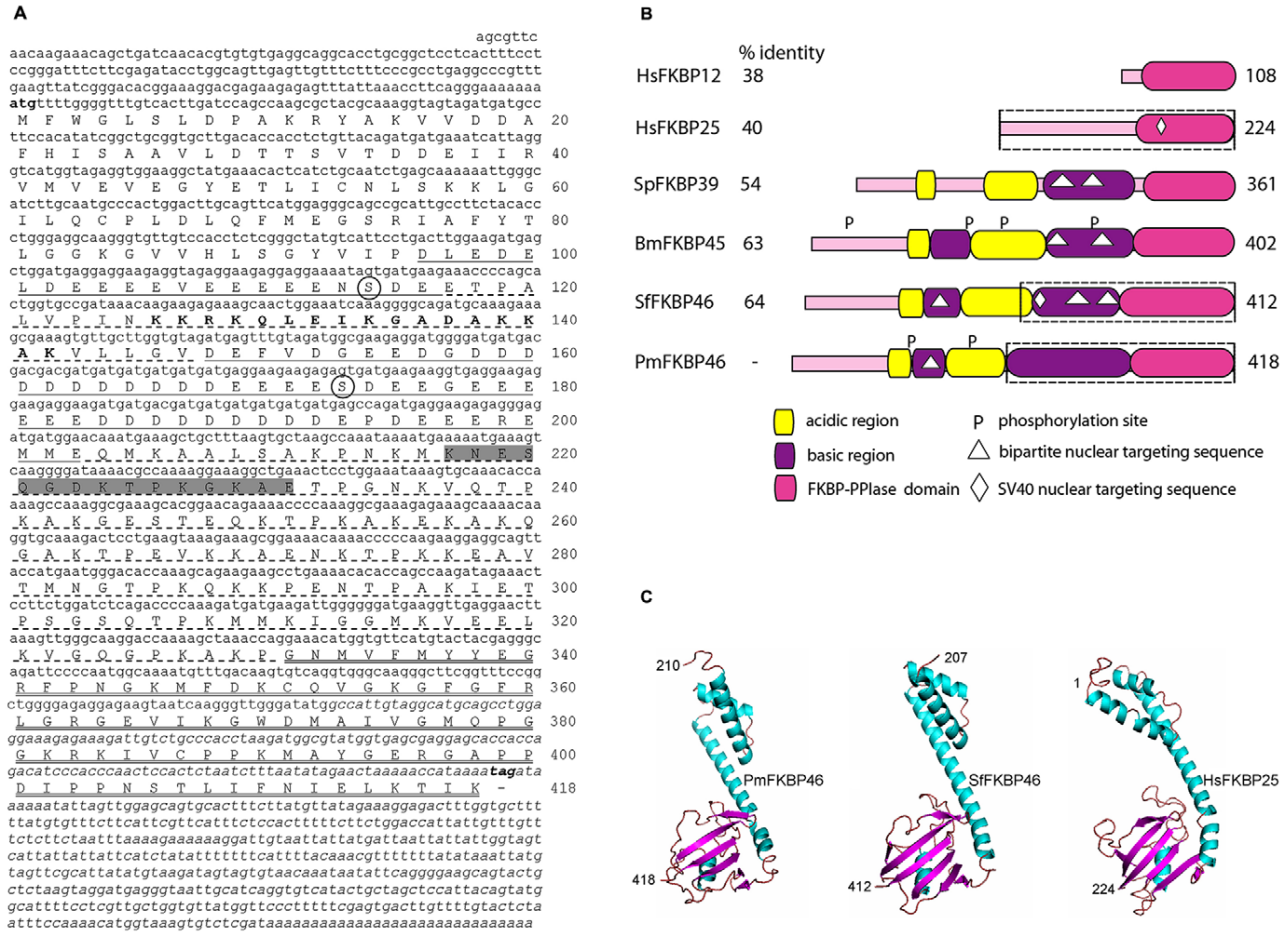
Sequence analysis of *PmFKBP46*. (**A**) The ORF of *PmFKBP46* consisted of 1,257 bp encoding 418 deduced amino acid residues. Two acidic regions containing the stretches of Asp and Glu-rich residues are indicated by a solid underline while two basic regions are indicated by a dashed underline. The bold capital letter sequence represents a bipartite nuclear targeting signal. The putative FKBP-PPIase domain at the C-termius is double underlined. The predicted phosphorylation sites are circled. The italicized nucleotide sequence indicates the yeast-two-hybrid identified fragment. The peptide used for antibody production is highlighted in grey. (**B**) The predicted PmFKBP46 schematic structure was compared with 4 nuclear-type FKBP proteins including human FKBP25 (HsFKBP25, accession number **Q00688**), fission yeast FKBP39 (SpFKBP39, accession number **O74191**), silkmoth FKBP45 (BmFKBP45, accession number **NP_001037356**), fall armyworm FKBP46 (SfFKBP46, accession number **Q26486**) and 1 cytosolic-type human FKBP12 (HsFKBP12, accession number **P62942**). The length of amino acid sequence is indicated on the right end. The % identity of the putative FKBP-PPIase domain of PmFKBP46 to that of other sequences is indicated on the left end. The dash boxes represent the fragments used for 3D model prediction. (**C**) The predicted 3D models of the C-terminal PmFKBP46, C-terminal SfFKBP46, and full-length HsFKBP25 were generated with the I-TASSER server. Numbers represent amino acid residues.

The full-length of *PmFKBP46* subsequently obtained by 5′RACE assays comprised a 5′ untranslated region (UTR) of 187 bp, an open reading frame (ORF) of 1,257 bp that encoded 418 deduced amino acid residues and a 3′ UTR of 482 bp (Fig. 2A). The sequence was deposited in the GenBank database under accession number **HQ191477**. The estimated molecular weight was approximately 46.3 kDa and the isoelectric point 4.58. BLASTP analysis of the entire deduced protein sequence showed high homology to immunophilin FKBP-like proteins of many insects including *S. frugiperda*, *Acyrthosiphon pisum* (accession number **XP_001951061**), *Bombyx mori* (accession number **NP_001037356**), *Drosophila mojavensis* (accession number **XP_002001540**) and *Apis mellifera* (accession number **XP_001121759**) with ranges of 37–63% identity and 55–75% similarity. FKBP proteins are a class of peptidyl-prolyl cis/trans isomerase (PPIase) enzymes first identified from the bovine thymus and human spleen as binding proteins of the immunosuppressive drug FK506 (also called Tacrolimus or Fujimycin) [30]. Proteins that bind to immunosuppressive drugs are called immunophilins. A BLASTP analysis restricted to crustaceans yielded a 39 kDa FK506-binding nuclear protein of *Lepeophtheirus salmonis* (salmon louse, accession number **ADD24248**). Based on sequence similarities and the predicted molecular mass, the newly identified cDNA sequence was designated *PmFKBP46*. Primary structural analysis of the PmFKBP46 protein revealed a conserved FKBP-PPIase domain at the C-terminus (amino acid residues 331–418) (Fig. 2A, B). In addition, characteristic signatures of nuclear-type FKBP proteins described previously were shown. These included two acidic regions (amino acid residues 96–117 and 148–203) and two basic regions (amino acid residues 118–147 and 204–330) (Fig. 2A, B). The bipartite nuclear targeting signal [31] was also identified within the first basic region (Fig. 2A, B). Putative phosphorylation sites were predicted at Ser^114^ and Ser^173^. The overall schematic structure of PmFKBP46 is displayed in Fig. 2B in comparison with 3 nuclear-type FKBP sequences from the fission yeast, silk moth and fall armyworm. Also included are one nuclear-type and one cytosolic-type of FKBP from humans. It was clearly revealed that the predicted nuclear targeting sequences were found in all the nuclear-type FKBP proteins, including PmFKBP46, but not in the cytosolic-type HsFKBP12. However, the PmFKBP46 sequence pattern was more similar to those of insects and yeast because it contained 2 acidic and 2 basic regions. The FKBP-PPIase domain of the PmFKBP46 sequence showed 38–64% identity to those of the other organisms analyzed (Fig. 2B).

In addition to primary structure, the 3D-structure of PmFKBP46 was predicted using the I-TASSER server. The full-length of the putative PmFKBP46 amino acid sequence yielded a negative C-score and was not subjected to further analysis. However, the C-terminal half of the sequence yielded a C-score of 0.84 indicating relatively higher reliability for the structure shown in Fig. 2C. The 3D-structures of the C-terminal half of the fall armyworm SfFKBP46 (C-score  = 1.14) and that of the full-length human FKBP25 (C-score  = 0.1) are also included in Fig. 2C. The overall structures shared significant similarity in their FKBP-PPIase domains with respect to 4–5 β-sheet strands and a helix-loop-helix (HLH) motif considered to be a nucleic acid binding region as previously reported in FKBP proteins [32], [33], [34].

### Confirmation of PmFKBP46 and VP15 interaction by GST pull-down assay

**Figure 3:**
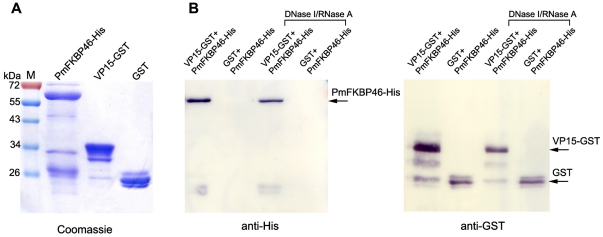
Interaction between PmFKBP46 and VP15 by GST pull-down assay. (**A**) Purified recombinant His-tagged or GST-tagged proteins produced in *E. coli* are analyzed by SDS-PAGE and Coomassie staining. (**B**) GST pull-down assay between purified PmFKBP46-His and VP15-GST proteins. PmFKBP46-His was incubated with VP15-GST or GST coupled with glutathione sepharose beads and the pull-down products were detected by anti-His or anti-GST antibodies as indicated by arrows. Nuclease-treated proteins were also used for pull-down assay to confirm the direct interaction between PmFKBP46 and VP15. M, pre-strained protein molecular weight markers (Fermentas).

Full-length sequences of *VP15* and *PmFKBP46* were cloned into plasmids to produce GST-tagged and His-tagged proteins, respectively. The purified VP15-GST had the predicted size of approximately 41 kDa (15 kDa +26 kDa of GST) while the size of PmFKBP46-His was ∼60 kDa which was larger than its predicted molecular weight of 48 kDa (46 kDa + 6x His epitope) (Fig. 3A). Interaction between PmFKBP46-His and VP15-GST was confirmed by pull-down assay using glutathione sepharose beads. The PmFKBP46-His was incubated with VP15-GST or GST (as negative control) which were pre-coupled with glutathione sepharose beads. After the beads were washed several times to remove unbound proteins, the protein complexes were analyzed by immunoblot assay detected with a mouse anti-His antibody and HRP-conjugated anti-GST antibody. It was shown that PmFKBP46-His was pulled-down by VP15-GST but not by GST (Fig. 3B). Since PmFKBP46 and VP15 posses DNA binding activity (described below), the pull-down assay was independently conducted with nuclease-treated proteins in order to determine whether the interaction between the two proteins occurred through nucleic acids. As shown in Fig. 3B (DNase I/RNase A panel), the binding between PmFKBP46-His and VP15-GST was still occurred in the absence of nucleic acids, indicating direct physical interaction.

### Custom-made antibody specific to PmFKBP46 in shrimp hemocytes

**Figure 4:**
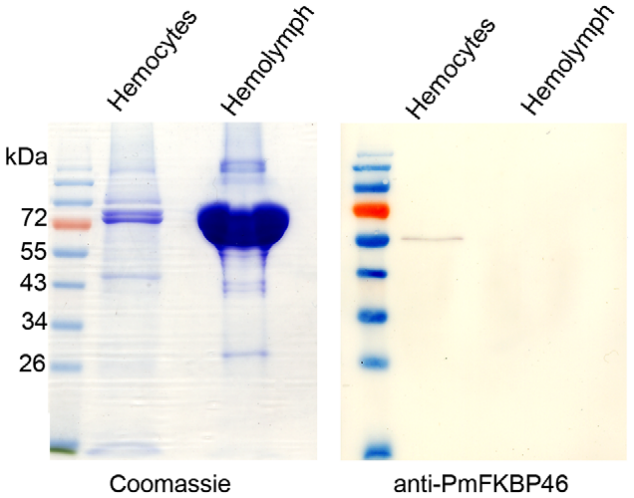
Specificity test for the PmFKBP46 antibody. Immunoblots of shrimp hemocyte lysate and hemolymph using a custom-made antibody against the PmFKBP46 peptide revealed the presence of detectable PmFKBP46 only in the hemocyte lysate.

The specificity of the custom-made mouse polyclonal antibody against the peptide of PmFKBP46 (amino acid residues 217-231) was tested by western blot using protein lysates from *P. monodon* hemolymph and hemocyte extracts. The antibody reacted specifically to a band around 55-60 kDa from shrimp hemocytes but showed no reactivity to bands from hemolymph (Fig. 4). The results indicated that the custom-made antibody was specific and suitable for use in co-localization assays. Note that the molecular mass of PmFKBP46 protein expressed in *E. coli* (results above) and in shrimp hemocytes (this study) were all higher than the estimated molecular weight (∼46 kDa) based on its deduced amino acid sequence, suggesting some posttranslational modification and/or highly charged nature of the protein. PmFKBP46 protein was also detected in shrimp gill lysates (data not shown). A transcription expression study by RT-PCR also indicated the presence of *PmFKBP46* transcripts in shrimp hemocytes and gills as well as other tested organs such as the hepatopancreas, intestine, lymphoid organ and stomach (Fig. S1).

### Co-localization of PmFKBP46 and VP15 in shrimp hemocytes

**Figure 5:**
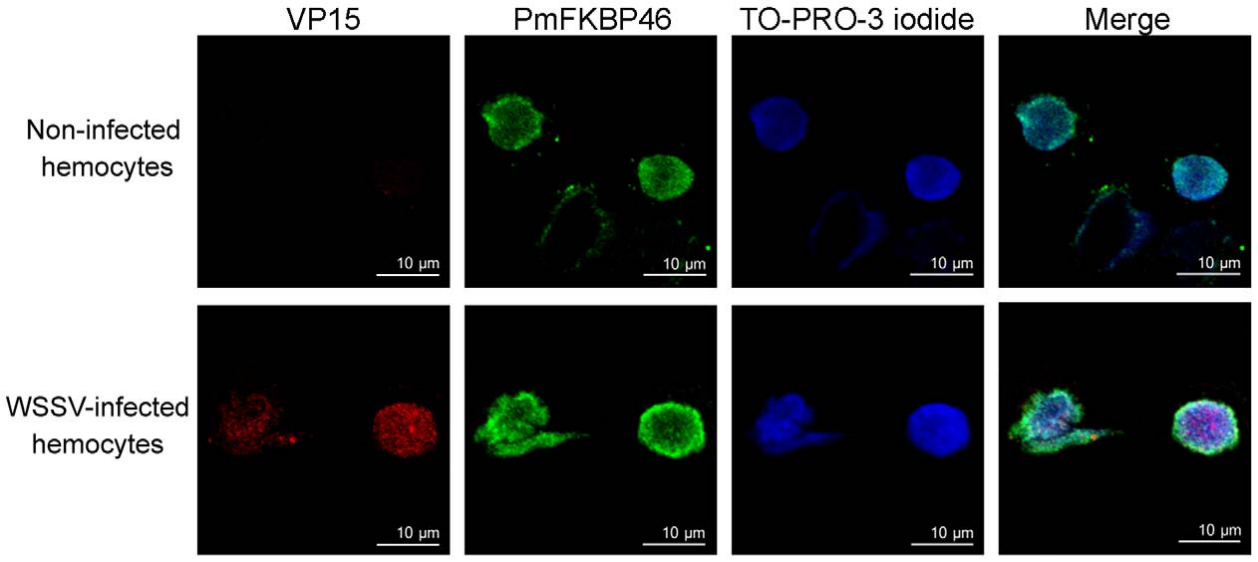
Both VP15 and PmFKBP46 present in the nucleus of WSSV-infected hemocytes. Hemocytes from normal and WSSV-infected shrimp (72 h p.i.) were analyzed by indirect immunofluoresence microscopy. In non-infected hemocytes, PmFKBP46 was visualized (green signal) with a mouse anti-PmFKBP46 antibody and FITC-conjugated goat anti-mouse IgG antibody. In WSSV-infected hemocytes, VP15 was visualized (red signal) with a rabbit anti-VP15 antibody and Cy3-conjugated goat anti-rabbit IgG antibody. Nuclei were visualized by counterstaining with TO-PRO-3 iodide (blue).

To add more evidence supporting their interaction, the location of PmFKBP46 and VP15 was investigated by indirect immunofluorescence assay in healthy and WSSV-infected shrimp (72 h p.i.). *In situ* immunocytochemistry by confocal microscopy using anti-VP15 and anti-PmFKBP46 antibodies (Fig. 5 upper panel), revealed that fluorescence for PmFKBP46 was evenly distributed in the nuclei of normal hemocytes while there was no fluorescence for VP15. By contrast (Fig. 5, lower panel), PmFKBP46 and VP15 were co-localized in the nuclei of hemocytes from WSSV-infected shrimp. In addition, the PmFKBP46 signal intensity appeared to be slightly increased in the presence of WSSV. Control staining experiments performed using i) preimmune serum alone, ii) each primary antibody alone, and iii) secondary antibodies alone did not yield any signal in the hemocytes, excluding the possibility of cross-reactivity between tested antibodies and hemocyte proteins (Fig. S2). Similar immunocytochemistry tests performed using Sf-9 cells cotransfected with PmFKBP46-V5 and VP15-FLAG recombinant plasmids also revealed both proteins co-localized in the nuclei (data not shown). All these experimental results suggested that VP15 and PmFKBP46 shared the same subcellular location both *in vitro* and *in vivo* and supported the proposal that interaction between PmFKBP46 and VP15 was genuine.

### Characteristics of DNA-binding by PmFKBP46 and VP15

**Figure 6:**
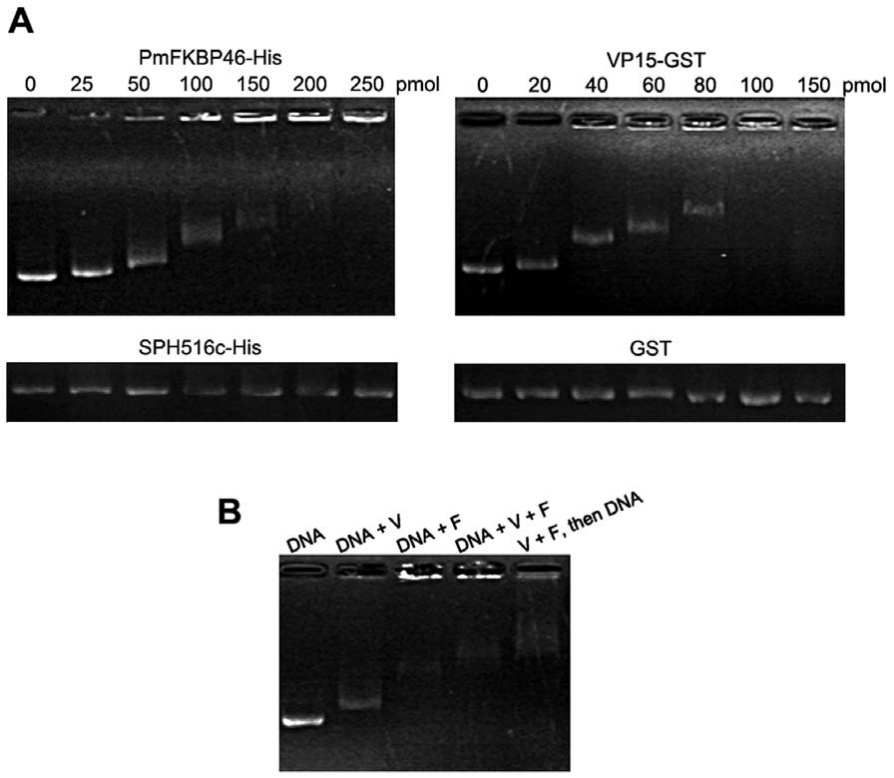
DNA-binding assay. (**A**) DNA-binding activity was investigated by electrophoretic mobility shift assay (EMSA). Plasmid DNA pGEX-5X-1 at 300 ng was incubated with 0–150 or 0–250 pmol of indicated recombinant proteins and subjected to agarose gel electrophoresis. (**B**) DNA binding activity of VP15-GST (V) and PmFKBP46-His (F) was investigated. Reactions containing individual proteins incubated with plasmid DNA pGEX-5X-1 are indicated as “DNA+V” and “DNA+F” while a control reaction with plasmid DNA alone is indicated as “DNA”. “DNA+V+F” indicates a reaction in which DNA and both proteins were incubated together at the same time. “V+F, then DNA” means the two proteins were incubated together prior to DNA addition.

The nuclear localization of PmFKBP46 (Fig. 5) and the presence of a putative helix-loop-helix in the deduced protein sequence (Fig. 2C) raised the possibility that it might have DNA binding activity similar to that previously reported for VP15 [17], [20]. Thus, electrophoretic mobility shift assays (EMSA) were carried out using plasmid DNA mixed with recombinant PmFKBP46-His or VP15-GST or with both and using recombinant SPH516c-His and GST as negative controls (Fig. 6A). Independent tests with increasing amounts of each protein (0–150 pmol or 0–250 pmol) revealed that DNA mobility was increasingly retarded (indicating binding) in a dose-dependent manner with PmFKBP46-His and VP15-GST but not with SPH516c-His or GST (Fig. 6A). At high concentrations of PmFKBP46-His and VP15-GST, a portion of the plasmid DNA remained, almost “immobilized” near the gel slot (Fig. 6A).

To test the single and combined binding activity of PmFKBP46-His (designated F) and VP15-GST (designated V), mobility assays were carried out using the separate proteins and the proteins mixed simultaneously or sequentially with the plasmid DNA (Fig. 6B). In lane “DNA+V” the migration of DNA was retarded when compared to the plasmid control (lane “DNA”) and some immobilized DNA was found near the gel slot. Using “DNA+F”, an increase in retardation was observed, and much of the DNA was immobilized. Moreover, two other treatments revealed that the presence of both V and F proteins in the reactions increased DNA retardation. It was found that DNA migration in “DNA+V+F” was slower than that in “DNA+V” and “DNA+F”. In addition, the result for “V+F, then DNA” (i.e., incubation of V+F prior to addition of DNA) gave the highest retardation of all the other treatments, suggesting that prior protein-protein binding improved DNA binding activity. A separate experiment was carried out with a different molar ratio of tested proteins and similar results were still observed (Fig. S3).

## Discussion

We employed a yeast two hybrid assay using VP15 nucleocapsid protein of WSSV as bait and identified a novel shrimp protein PmFKBP46 as a putative binding partner of VP15. Although yeast co-transformed between empty vector pGBKT7 and rescued library AD-PmFKBP46c plasmid yielded a false positive interaction, other independent experiments were carried out to verify the protein interaction. We have shown that full-length PmFKBP46 and VP15 bind together by GST pull-down assay and by a co-localization assay in hemocyte nuclei of shrimp infected with WSSV. The reason why AD-PmFKBP46c activated the reporter genes might be due to the conserved FKBP-PPIase domain at the PmFKBP46c C-terminus (i.e., homologous to FKBP12) that has been reported to be involved in transcriptional activities (See review by [35]). It was found that FKBP12 interacted with transcription factor YY1 [36] that could, in turn, activate transcription of the target promoter [37]. By electrophoretic mobility shift assays (EMSA), we confirmed DNA binding by VP15 as previously reported [17], [20] and showed that PmFKBP46 also binds with DNA, as was suggested by the putative helix-loop-helix structure predicted from its deduced amino acid sequence.

With respect to the significance of binding between PmFKBP46 and VP15 in terms of WSSV virion production in host cells, there is still too little information for firm conclusions. The higher intensity of fluorescence by immunocytochemistry for PmFKBP46 in WSSV-infected nuclei than in normal nuclei, might suggest that WSSV recruits PmFKBP46 protein for its genome packaging process or that VP15 is transported to the nucleus by the nuclear targeting capability of PmFKBP46. In a previous report, VP15 was proposed to contain a nuclear targeting sequence necessary for nuclear translocation [38], but the results as reported would have been the same if VP15 transport depended on its specific binding to a host protein that targeted the nucleus, as speculated here. Further work is needed to determine the actual situation, but it is notable that the nuclear targeting motif in PmFKBP46 more clearly resembles the model motif than that of VP15. It would also be interesting to investigate whether other WSSV proteins also bind to PmFKBP46. In any case, there is no evidence from published reports that PmFKBP46 is among the 40 proteins reported to be present in the WSSV virion [39], so even though it binds specifically to VP15 and may be involved in VP15 transport to the nucleus, it must be separated from VP15 during virion assembly. This might occur during the formation of the fibrillar complex of DNA and viral proteins that has been reported to occur prior to DNA packaging in the WSSV nucleocapsid [27]. Such hypotheses could be tested using immunocytochemical analysis of VP15 distribution after PmFKBP46 knockdown or immunogold assays by electron microscopy to examine the ultrastructural locations of PmFKBP46 and VP15 during virion assembly.

Also requiring an explanation is the molecular weight discrepancy between the deduced mass of PmFKBP46 and the expressed proteins from shrimp hemocytes and from the bacterial system. Such a discrepancy has been reported also in the silkmoth FKBP45 [34] and in human FKBP25 [40]. The apparent mass difference might be due to not only posttranslational size alteration but also to charge modification, especially with respect to multiple charged regions present in the predicted primary structure.

FKBP proteins have been reported from various subcellular locations such as the cytosol, nuclei, chloroplasts, and the endoplasmic reticulum [41], [42], [43], indicating family involvement in multiple cellular processes. Accordingly, diverse binding proteins and chemical molecules have been found to interact with FKBP proteins. For example, human FKBP38 interacts with Bcl-2 and Bcl-X_L_ resulting in cell protection against apoptosis [44], [45]. Also, the first-discovered binding molecules of FKBP were the immunosuppressive drugs FK506 (tacrolimus) and rapamycin (sirolimus) [30], [46], [47]. The FKBP-drug complexes interfere with a variety of signal transduction pathways leading to immunosuppression (See review by [48]). Interaction between viruses and FKBP proteins has also been reported. For example, it was reported that human FKBP8 binds to a nonstructural protein 5A replicase of hepatitis C virus in a manner essential for viral RNA replication [49]. Interestingly, FKBP12 protein was found to reside within the human immunodeficiency virus type 1 (HIV-1) virion, although the functional significance of this is still under investigation (see review by [50]). As stated above, there does not seem to be any indication that PmFKBP46 is incorporated in the WSSV virion, but negative results from a simple test using anti-PmFKBP46 antibody in a western blot of proteins from purified WSSV particles would confirm that contention.

Apart from any proposed role in interacting with WSSV, the normal biological function of PmFKBP46 in shrimp also remains to be defined. Functional properties of homologous FKBP in the fall armyworm and silkmoth models are also obscure and most information on FKBP proteins has been gained from research on yeasts, plants, and humans. For example, human nuclear FKBP25 was shown to be involved in regulating gene expression by interaction with transcription factor YY1 and is also associated with histone deacetylases [51]. Nuclear FKBP39 of yeast *S. pombe* was found to have capacity for nucleosome assembly [52]. Since FKBP proteins exhibit a high degree of structural conservation, related fundamental roles of PmFKBP46 in shrimp might be expected.

In conclusion, we have identified a novel protein of the FKBP family in shrimp and revealed its ability to bind with and co-interact with WSSV VP15 in DNA-binding, but the significance of this interaction in terms of the WSSV replication cycle requires further investigation.

## Supporting Information

Figure S1 **Tissue distribution analysis of** ***PmFKBP46*** **.** Expression of *PmFKBP46* in various tissues of *P. monodon* was analyzed by RT-PCR. *β-actin* was used as an internal control and amplified in separate reactions but loaded in the same well as the *PmFKBP46* products from each respective sample. GL, gills; HC, hemocytes; HP, hepatopancreas; IN, intestine; LO, lymphoid organ; ST, stomach.
(DOC)

Figure S2 **Control staining of WSSV-infected hemocytes in the co-localization experiment of PmFKBP46 and VP15.** In Row 1, hemocytes were incubated with preimmune serum followed by staining with secondary antibodies both FITC-conjugated goat anti-mouse IgG antibody (green signal) and Cy3-conjugated goat anti-rabbit IgG antibody (red signal). In Row 2, hemocytes were stained only with primary antibodies (mouse anti-PmFKBP46 antibody or rabbit anti-VP15 antibody). In Row 3, hemocytes were stained only with secondary antibodies (FITC-conjugated goat anti-mouse IgG antibody or Cy3-conjugated goat anti-rabbit IgG antibody). Nuclei were visualized by counterstaining with TO-PRO-3 iodide (blue signal).
(DOC)

Figure S3 **DNA-binding assay.** DNA binding activity of VP15-GST (V) and PmFKBP46-His (F) was investigated using 1∶2 molar ratio containing 40 pmol of V and 80 pmol of F proteins. Reactions containing individual proteins incubated with plasmid DNA pGEX-5X-1 are indicated as “DNA+V” and “DNA+F” while a control reaction with plasmid DNA alone is indicated as “DNA”. “DNA+V+F” indicates a reaction in which DNA and both proteins were incubated together at the same time. “V+F, then DNA” means the two proteins were incubated together prior to DNA addition.
(DOC)
