# Supplementary material for: Co-Interactive DNA-Binding between a Novel, Immunophilin-Like Shrimp Protein and VP15 Nucleocapsid Protein of White Spot Syndrome Virus
Source: PLoS One. 2011 Sep 29;6(9):e25420. doi: 10.1371/journal.pone.0025420 (PMC3183051; doi:10.1371/journal.pone.0025420)
Supplement: Figure S1 — Tissue distribution analysis of PmFKBP46 . Expression of PmFKBP46 in various tissues of P. monodon was analyzed by RT-PCR. β-actin was used as an internal control and amplified in separate reactions but loaded in the same well as the PmFKBP46 products from each respective sample. GL, gills; HC, hemocytes; HP, hepatopancreas; IN, intestine; LO, lymphoid organ; ST, stomach. (DOC) [file pone.0025420.s001.doc]

**Supporting Information**


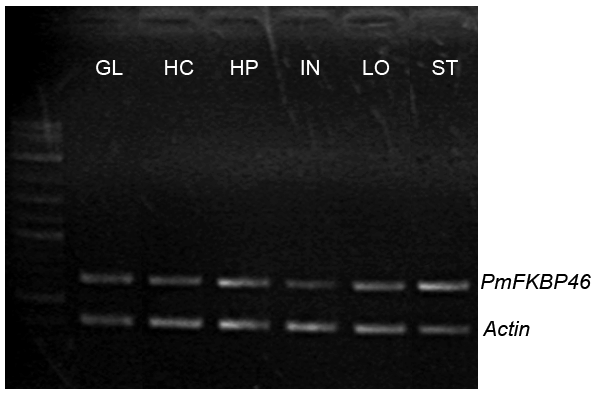


**Supplemental Figure S1. Tissue distribution analysis of *PmFKBP46***

Expression of *PmFKBP46* in various tissues of *P. monodon* was analyzed by RT-PCR. *β-actin* was used as an internal control and amplified in separate reactions but loaded in the same well as the *PmFKBP46* products from each respective sample. GL, gills; HC, hemocytes; HP, hepatopancreas; IN, intestine; LO, lymphoid organ; ST, stomach.
