# Supplementary material for: Co-Interactive DNA-Binding between a Novel, Immunophilin-Like Shrimp Protein and VP15 Nucleocapsid Protein of White Spot Syndrome Virus
Source: PLoS One. 2011 Sep 29;6(9):e25420. doi: 10.1371/journal.pone.0025420 (PMC3183051; doi:10.1371/journal.pone.0025420)
Supplement: Figure S2 — Control staining of WSSV-infected hemocytes in the co-localization experiment of PmFKBP46 and VP15. In Row 1, hemocytes were incubated with preimmune serum followed by staining with secondary antibodies both FITC-conjugated goat anti-mouse IgG antibody (green signal) and Cy3-conjugated goat anti-rabbit IgG antibody (red signal). In Row 2, hemocytes were stained only with primary antibodies (mouse anti-PmFKBP46 antibody or rabbit anti-VP15 antibody). In Row 3, hemocytes were stained only with secondary antibodies (FITC-conjugated goat anti-mouse IgG antibody or Cy3-conjugated goat anti-rabbit IgG antibody). Nuclei were visualized by counterstaining with TO-PRO-3 iodide (blue signal). (DOC) [file pone.0025420.s002.doc]

**Supporting Information**

**
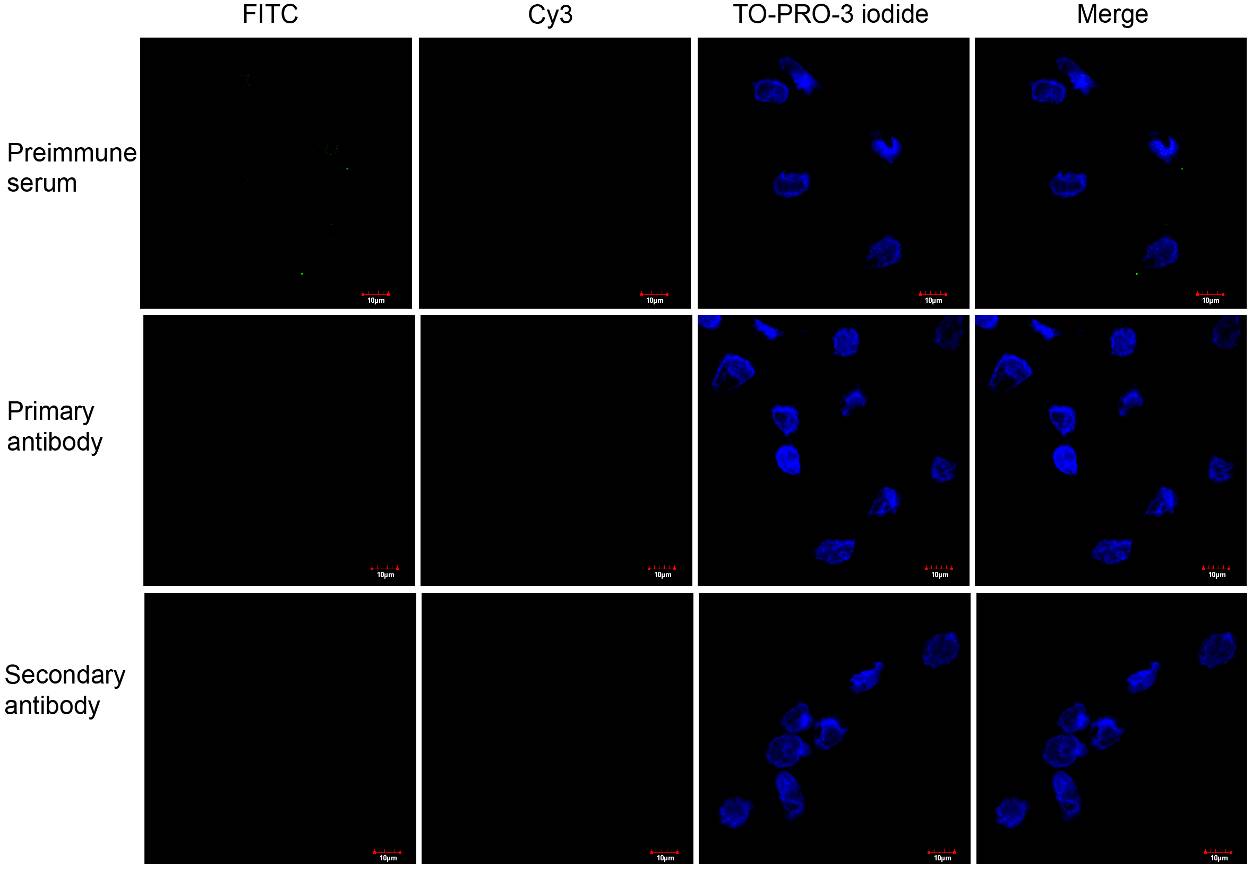
**

**Supplemental Figure S2. Control staining of WSSV-infected hemocytes in the co-localization experiment of PmFKBP46 and VP15**

In row 1, hemocytes were incubated with preimmune serum followed by staining of secondary antibodies both FITC-conjugated goat anti-mouse IgG antibody (green signal) and Cy3-conjugated goat anti-rabbit IgG antibody (red signal). In row 2, hemocytes were stained only with primary antibodies (mouse anti-PmFKBP46 antibody or rabbit anti-VP15 antibody). In row 3, hemocytes were stained only with secondary antibodies (FITC-conjugated goat anti-mouse IgG antibody or Cy3-conjugated goat anti-rabbit IgG antibody). Nuclei were visualized by counterstaining with TO-PRO-3 iodide (blue signal).
