# Supplementary material for: Co-Interactive DNA-Binding between a Novel, Immunophilin-Like Shrimp Protein and VP15 Nucleocapsid Protein of White Spot Syndrome Virus
Source: PLoS One. 2011 Sep 29;6(9):e25420. doi: 10.1371/journal.pone.0025420 (PMC3183051; doi:10.1371/journal.pone.0025420)
Supplement: Figure S3 — DNA-binding assay. DNA binding activity of VP15-GST (V) and PmFKBP46-His (F) was investigated using 1∶2 molar ratio containing 40 pmol of V and 80 pmol of F proteins. Reactions containing individual proteins incubated with plasmid DNA pGEX-5X-1 are indicated as “DNA+V” and “DNA+F” while a control reaction with plasmid DNA alone is indicated as “DNA”. “DNA+V+F” indicates a reaction in which DNA and both proteins were incubated together at the same time. “V+F, then DNA” means the two proteins were incubated together prior to DNA addition. (DOC) [file pone.0025420.s003.doc]

**Supporting Information**


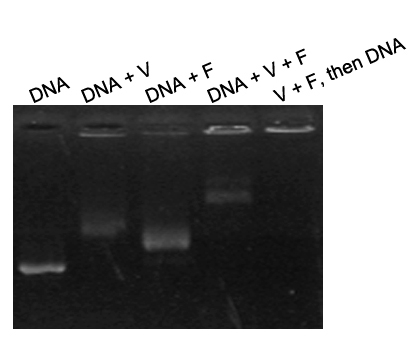


**Supplemental Figure S3. DNA-binding assay**

DNA binding activity of VP15-GST (V) and PmFKBP46-His (F) was investigated using 1:2 molar ratio containing 40 pmol of V and 80 pmol of F proteins. Reactions containing individual proteins incubated with plasmid DNA pGEX-5X-1 are indicated as “DNA+V” and “DNA+F” while a control reaction with plasmid DNA alone is indicated as “DNA”. “DNA+V+F” indicates a reaction in which DNA and both proteins were incubated together at the same time. “V+F, then DNA” means the two proteins were incubated together prior to DNA addition.
